# A Protective Lipidomic Biosignature Associated with a Balanced Omega-6/Omega-3 Ratio in *fat-1* Transgenic Mice

**DOI:** 10.1371/journal.pone.0096221

**Published:** 2014-04-23

**Authors:** Giuseppe Astarita, Jennifer H. McKenzie, Bin Wang, Katrin Strassburg, Angela Doneanu, Jay Johnson, Andrew Baker, Thomas Hankemeier, James Murphy, Rob J. Vreeken, James Langridge, Jing X. Kang

**Affiliations:** 1 Health Sciences, Waters Corporation, Milford, Massachusetts, United States of America; 2 Department of Biochemistry and Molecular & Cellular Biology, Georgetown University, Washington, DC, United States of America; 3 Laboratory for Lipid Medicine and Technology, Department of Medicine, Massachusetts General Hospital and Harvard Medical School, Boston, Massachusetts, United States of America; 4 Analytical Biosciences, Leiden Academic Centre for Drug Research, Leiden University, Leiden, The Netherlands; 5 Netherlands Metabolomics Centre, Leiden University, Leiden, The Netherlands; Scottish Association for Marine Science, United Kingdom

## Abstract

A balanced omega-6/omega-3 polyunsaturated fatty acid (PUFA) ratio has been linked to health benefits and the prevention of many chronic diseases. Current dietary intervention studies with different sources of omega-3 fatty acids (omega-3) lack appropriate control diets and carry many other confounding factors derived from genetic and environmental variability. In our study, we used the *fat-1* transgenic mouse model as a proxy for long-term omega-3 supplementation to determine, in a well-controlled manner, the molecular phenotype associated with a balanced omega-6/omega-3 ratio. The *fat-1* mouse can convert omega-6 to omega-3 PUFAs, which protect against a wide variety of diseases including chronic inflammatory diseases and cancer. Both wild-type (WT) and *fat-1* mice were subjected to an identical diet containing 10% corn oil, which has a high omega-6 content similar to that of the Western diet, for a six-month duration. We used a multi-platform lipidomic approach to compare the plasma lipidome between *fat-1* and WT mice. In fat-1 mice, an unbiased profiling showed a significant increase in the levels of unesterified eicosapentaenoic acid (EPA), EPA-containing cholesteryl ester, and omega-3 lysophosphospholipids. The increase in omega-3 lipids is accompanied by a significant reduction in omega-6 unesterified docosapentaenoic acid (omega-6 DPA) and DPA-containing cholesteryl ester as well as omega-6 phospholipids and triacylglycerides. Targeted lipidomics profiling highlighted a remarkable increase in EPA-derived diols and epoxides formed via the cytochrome P450 (CYP450) pathway in the plasma of *fat-1* mice compared with WT mice. Integration of the results of untargeted and targeted analyses has identified a lipidomic biosignature that may underlie the healthful phenotype associated with a balanced omega-6/omega-3 ratio, and can potentially be used as a circulating biomarker for monitoring the health status and the efficacy of omega-3 intervention in humans.

## Introduction

**Figure 1:**
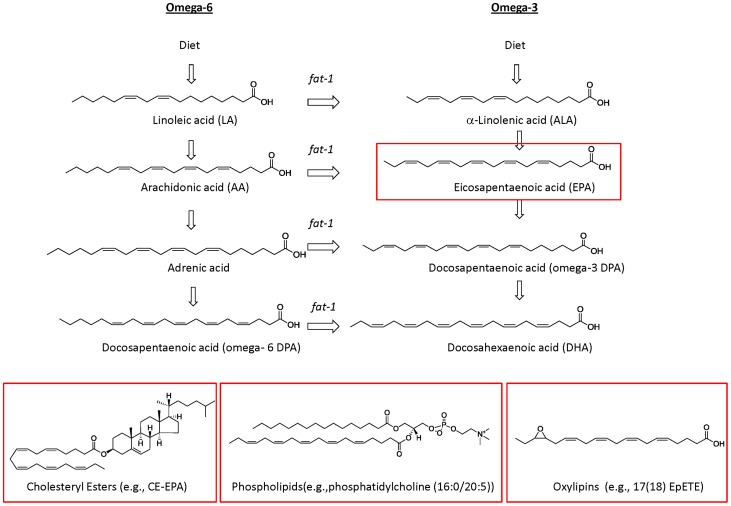
Overview of the omega-6 and omega-3 PUFAs metabolism. Diet-derived omega-6 linoleic acid (LA) and omega-3 alpha-linolenic acid (ALA) are transformed into longer chains PUFAs by the sequential action of desaturases and elongases. PUFAs can be found in blood as unesterified fatty acids, esterified to more complex lipids such as cholesteryl esters and phospholipids, or converted into the oxygenated metabolites oxylipins. The figure shows chemical structures of fatty acids and derivatives highlighted in our study.

Most Western diets are deficient in omega-3 polyunsaturated fatty acids (PUFAs) and abundant in omega-6 PUFAs [1]. Current nutritional research shows that a diet enriched in omega-3s offers health benefits and anti-inflammatory properties and that an excess of omega-6s might contribute to the pathogenesis of many chronic diseases, including cardiovascular, autoimmune and Alzheimer's diseases [2]–[10]. The imbalance between omega-6s and omega-3s is largely the result of the traditional reliance of Western diets on vegetables oils such as corn, soybean, safflower, and sunflower. These oils are enriched in omega-6 PUFAs, such as linoleic acid (LA), which can be metabolized in animals and humans to form longer chain fatty acids such as di-homo-gamma-linolenic acid (DGLA), docosapentaenoic acid (omega-6 DPA), and arachidonic acid (AA) are (Figure 1). At the same time, Western diets are lacking in leafy green vegetables, which are enriched in the omega-3 fatty acid, alpha-linolenic acid (ALA), and in oily fish, which contain the longer-chain omega-3 PUFAs such as eicosapentaenoic acid (EPA), omega-3 DPA, and docosahexaenoic acid (DHA) (Figure 1).

The human body cannot synthesize PUFAs de novo and must rely entirely on dietary intake for these essential nutrients. Also, it cannot interconvert between omega-6 and omega-3 fatty acids. The PUFAs, once absorbed in the intestines, are then transported, via the bloodstream, to all tissues. They can be found as unesterified fatty acids or esterified to complex lipids (e.g., phospholipids, cholesteryl esters and triacylglycerols) and can be metabolized into bioactive species (e.g., oxylipins) (Figure 1). Hundreds of small molecules have been identified as metabolites of these few omega-3 and omega-6 precursors in human tissue. Yet, it is the overall balance between omega-3s and omega-6s that seems to modulate many biological processes including the relaxation and contraction of smooth muscle tissue, blood coagulation, and – significantly – inflammation [11]–[13].

Although much research demonstrates a potentially important relationship between PUFA intake and the risk of disease, it remains challenging in current dietary intervention studies to accurately evaluate the impact of increased intake of omega-3s by food or supplementation. A frequently confounding factor is the variability inherent in studies of control diets. A different nutritional value may accompany such diets compared with a diet enriched in omega-3– a different composition of fatty acids, for example. Other frequent issues relate to the chemical nature, source, and dose of the omega-3 used in the dietary intervention studies. These issues include the mixed use of the different types of omega-3s, such as EPA and DHA, or the different forms of omega-3s, such as triacylglycerols, phospholipids, or ethyl esters. Furthermore, dietary intervention studies in humans are often associated with high individual genetic and environmental variability [14]. All of these factors militate against an accurate evaluation of the biological effects of omega-3s, and no molecular markers of omega-3 intake currently exist.

In 2004, the *fat-1* transgenic mouse model was developed to eliminate many of the confounders inherent in omega-6/omega-3 research [15]. The mouse was engineered to carry the *C. elegans fat-1* gene, which can add a double bond into an unsaturated fatty-acid hydrocarbon chain, thus converting omega-6 to omega-3 fatty acids (Figure 1) [15]. Though the mice are not exposed to an omega-3 diet, this conversion results in an abundance of omega-3 and a reduction in omega-6 fatty acids in their organs. The resulting omega-6/omega-3 fatty acid profile has also been shown to be comparable to those obtained by dietary supplementation [16]. The animals therefore provide a controlled approach for evaluating the effects of a balanced omega-6/omega-3 ratio, one that does not introduce the confounding factors that result from enforcing different test diets. To date, the *fat-1* transgenic mouse model has been widely used, and has demonstrated that balancing the omega-6/omega-3 ratio can protect against a wide variety of diseases, including chronic inflammatory diseases and cancer [3]. However, the molecular mechanisms underlying these beneficial effects remain to be fully elucidated.

In the present study, we used a multi-platform lipidomic approach to compare the molecular phenotype of *fat-1* and WT mice exposed for six months to an identical high-omega-6 diet in order to identify the biosignature involved in the health benefits associated with a balanced omega-6/omega-3 tissue ratio.

## Materials and Methods

### Materials

All chemicals were purchased from Sigma-Aldrich (Seelze, Germany) and were of analytical grade or higher purity. Lipid standards were purchased from Avanti Polar Lipids (Alabaster, Alabama USA), Cayman Chemical (Ann Arbor, Michigan USA), Biomol (Plymouth Meeting, Pennsylvania USA), and Larodan Fine Chemicals (Malmö, Sweden). For solid-phase extraction, 96-well, Waters Oasis HLB plates (60 mg) were obtained from Waters Corporation (Milford, Massachusetts USA).

### Animals and sample collection

Transgenic *fat-1* C57BL/6J mice were generated as previously described [15]. Only heterozygous female mice, aged 6 months, were used in this study. The mice were fed an identical diet rich in omega-6 PUFA and low in omega-3 PUFA (Catalog #1812692, Test Diet, Richmond, Indiana USA), a modification of the Test Diet AIN-76A semi-purified diet 58B0, with 10% total corn oil. Both wild type and *fat-1* mice were fed the same diet since weaning until the mice were sacrificed. The diet components and fatty acid composition of the diet are listed in Table S1. The animals were phenotyped according to their omega-6 and omega-3 PUFA profiles, as determined by gas chromatography [17]. For the experiments described, WT and *fat-1* littermates were used (n = 5). At approximately 6 months of age, all mice underwent 18 hours of fasting and were afterward euthanized by means of carbon dioxide. Total blood volume was immediately collected using cardiac puncture, and the blood was retained on ice. Whole-blood samples were centrifuged at 1,000×g for 15 min at 4°C. Plasma was transferred to a clean polypropylene tube and centrifuged at 10,000×g for 10 min at 4°C. Aliquots of the samples were drawn and stored at −80°C until assayed. All animal procedures were performed in accordance with guidelines prescribed by the Massachusetts General Hospital (MGH) Animal Committee and with Institutional Animal Care and Use Committee (IACUC) for MGH approval (Protocol #2010N000038). All efforts possible were made to minimize animal suffering. Animal investigations conformed to the Public Health Policy on Humane Care and Use of Laboratory Animals.

### Lipid extraction from mouse plasma

Total lipids were extracted from 10 μL of plasma after protein precipitation with 490 μL of isopropanol (IPA) spiked with the following internal standard: d_8_ arachidonic acid, cholesterol-d_7_, C19∶0-cholesteryl ester, trinonadecenoin, 1,2-dimyristoyl-*sn*-glycero-3-phosphoethanolamine, 1,2-dimyristoyl*-sn*-glycero-3-phosphocholine, 1-heptadecenoyl-2-hydroxy-sn-glycero-3-phosphocholine. Samples were vortex mixed, incubated at 4°C for 30 min, and centrifuged at 10,000×g and 4°C for 10 min. Supernatant was then transferred to mass-spectrometry vials for further analysis. Extraction of oxylipins was performed as previously described [18]. Briefly, after thawing on ice, 50 μL of plasma samples were treated immediately with antioxidants (0.2 mg butylated hydroxytoluene (BHT/EDTA)) and spiked with the following internal standards: 6k-PGF_1α_-d_4_, TXB_2_-d_4_, PGF_2_α-d_4_, PGE2-d_4_, PGD_2_-d_4_, LTE_4_-d_3_, LTB_4_-d_4_, 12,13 diHOME-d_4_, 9,10-diHOME-d_4_, 14,15-DiHETrE-d11, 15-deoxy-δ-12,14-PGJ_2_-d_4_, 20-HETE-d_6_, 9S-HODE-d_4_, 12S-HETE-d_8_, 5S-HETE-d_8_, 5-oxo-ETE-d_7_. Plasma samples were loaded onto the solid-phase extraction plate and then eluted with 1.5 mL ethyl acetate, after wetting the plate wells with 0.5 mL methanol. The eluent was reduced under nitrogen and subsequently reconstituted in a 50-µL solution of methanol and acetonitrile (1∶1) containing 100 nM 1-cyclohexyluriedo-3-dodecanoic acid, a quality marker for the analysis.

### Liquid Chromatography Mass Spectrometry

Total lipid analysis was performed with IonKey/MS system comprised of an ACQUITY UPLC M-Class, the ionKey source and an iKey CSH C18 130 Å, 1.7 µm particle size, 150 μm ×100 mm column (Waters Corporation, Milford, Massachusetts, USA) coupled to a Synapt G2-S or Xevo TQ-S mass spectrometer (Waters Corporation, Manchester, UK). The capillary voltage was 2.8 kV and the source temperature 110°C. Injections were 0.5 μL using partial-loop mode with a column temperature of 55°C and flow rate of 3 µL/min. Mobile phase A consisted of acetonitrile/water (60/40) with 10 mM ammonium formate +0.1% formic acid. Mobile phase B consisted of IPA/acetonitrile (90/10) with 10 mM ammonium formate +0.1% formic acid. The gradient was programmed as follows: 0.0–2.0 min from 40% B to 43% B, 2.0–2.1 min to 50% B, 2.1–12.0 min to 99% B, 12.0–12.1 min to 40% B, and 12.1–14.0 min at 40% B. For the untargeted analyses, a mass range of 50–1500 *m/z* was selected in both positive and negative electrospray ionization.

Oxylipins were analyzed using UPLC (Waters Corporation, Milford, Massachusetts USA) coupled to electrospray ionization on a Xevo TQ-S mass spectrometer (Waters Corporation, Manchester, UK). The auto sampler was cooled to 10°C. For each analysis, 3 µL of sample was injected on a BEH C18 1.7 µm, 2.1×100 mm column (Waters Corporation, Milford, Massachusetts, USA) using a flow rate of 0.6 mL/min at 40°C. Mobile phases were composed as follows: A = 0.1% acetic acid, and B = 90∶10 v/v acetonitrile/IPA (0.00–1.00 min from 25% B to 33% B, 1.00–8.00 min B to 95%, 8.00–8.50 min to 95% B, 8.51–10.00 min to 25% B). Electrospray ionization was performed in the negative-ion mode applying a capillary voltage of 2 kV, a source temperature of 120°C, a desolvation gas temperature of 350°C, a desolvation gas flow of 650 L/h, a cone-gas flow of 150 L/h, a collision voltage of 15 V, and a cone voltage of 35 V. Oxylipins were detected using MRM transitions optimized using synthetic standards (Table S2). Limits of quantification were in the lower pM range.

### Data Processing and Analysis

For the untargeted lipidomic approach, a combination of analysis of the variance (ANOVA) and multivariate statistics, including principal component analysis (PCA) and partial, least-squares discriminant analysis (PLS-DA), identified lipids most responsible for differences between *fat-1* and WT sample groups. Compounds were identified by database searches as performed by Human Metabolome Database (HMDB) [19] and METLIN [20] as well as by fragmentation patterns, retention times and ion-mobility-derived collision cross sections versus commercially available reference standards, when available. For the targeted approach, mean concentrations and SEM values (n = 5) for each group (*fat-1* and WT) were calculated using appropriate internal standards to normalize for variations in sample preparation and MS detection. Univariate analyses (Student's *t*-tests) were conducted to assess for significance (p-value) and false-discovery rate (FDR) was used to control for multiple comparison. A volcano plot was produced to compare the fold-change versus the p-value for the targeted lipids.

Data processing and analysis was conducted using Progenesis QI Informatics (Nonlinear Dynamics, Newcastle, UK) and MetaboAnalyst 2.0 [21]. Data quantification was performed using TargetLynx software (Waters Corporation, Milford, Massachusetts USA).

## Results

**Figure 2:**
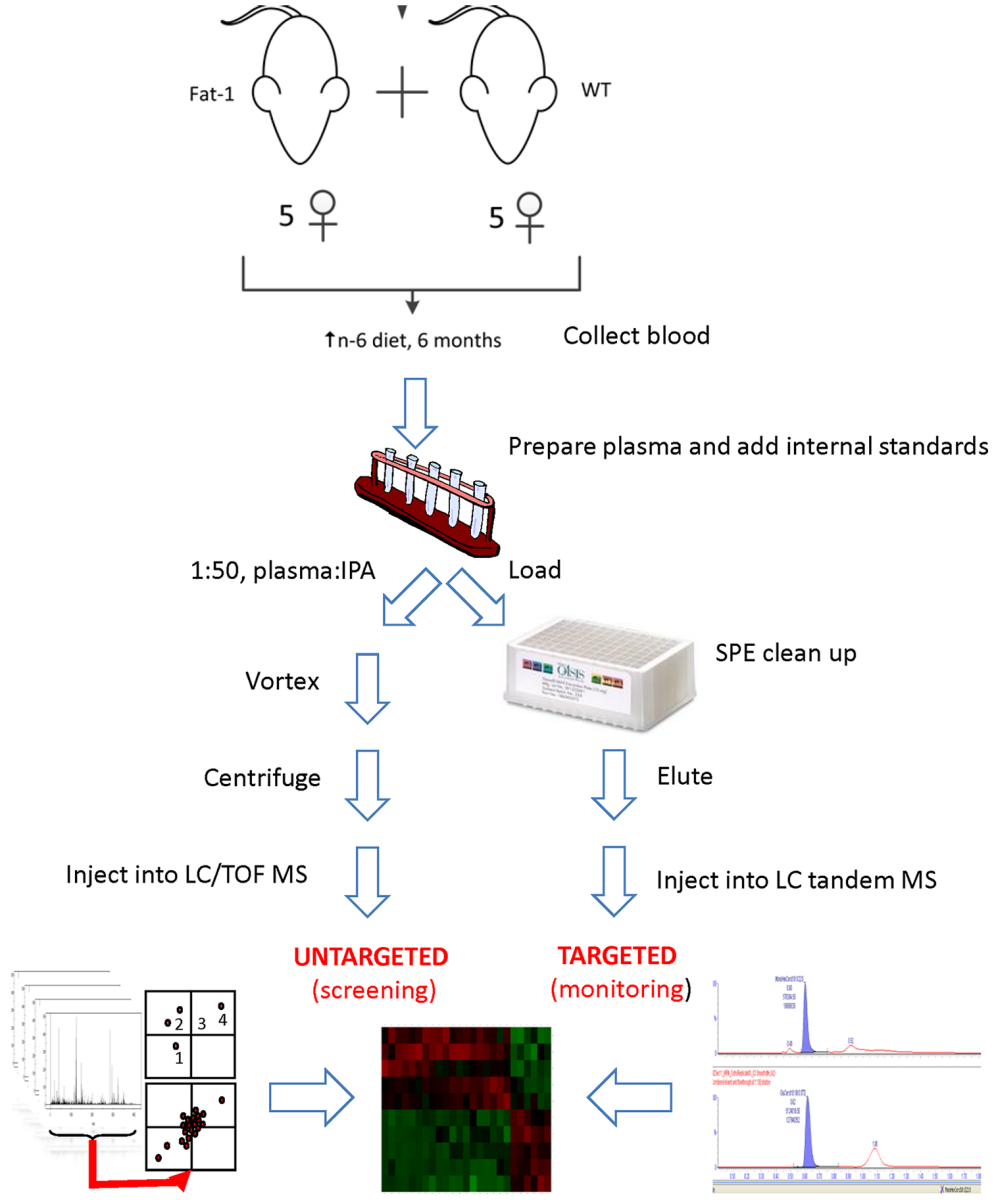
Study design and workflow for the lipidomic analyses. Heterozygous female *fat-1* and WT mice were fed a 6-month-long diet containing 10% corn oil, which is particularly enriched in omega-6 PUFAs. Blood was collected and plasma samples were prepared and divided into two aliquots. Before extraction, a mixture of internal standards was added to the plasma to normalize for variations in sample preparation or MS detection. Complementary untargeted and targeted lipidomic analyses were conducted, and the results were integrated for the generation of a unique lipidomic biosignature characteristic of the balanced omega-6/omega-3 ratio found in *fat-1* mice.

To characterize the molecular phenotype associated with a balanced omega-6/omega-3 ratio, a multi-platform lipidomic approach was used (Figure 2). Plasma samples from the fat-1 and WT groups were divided into two aliquots, one for an untargeted lipidomic approach to screen for major lipid alterations, and the other for a multiplexed targeted lipidomic approach to quantify low-abundance, bioactive lipid species.

**Figure 3:**
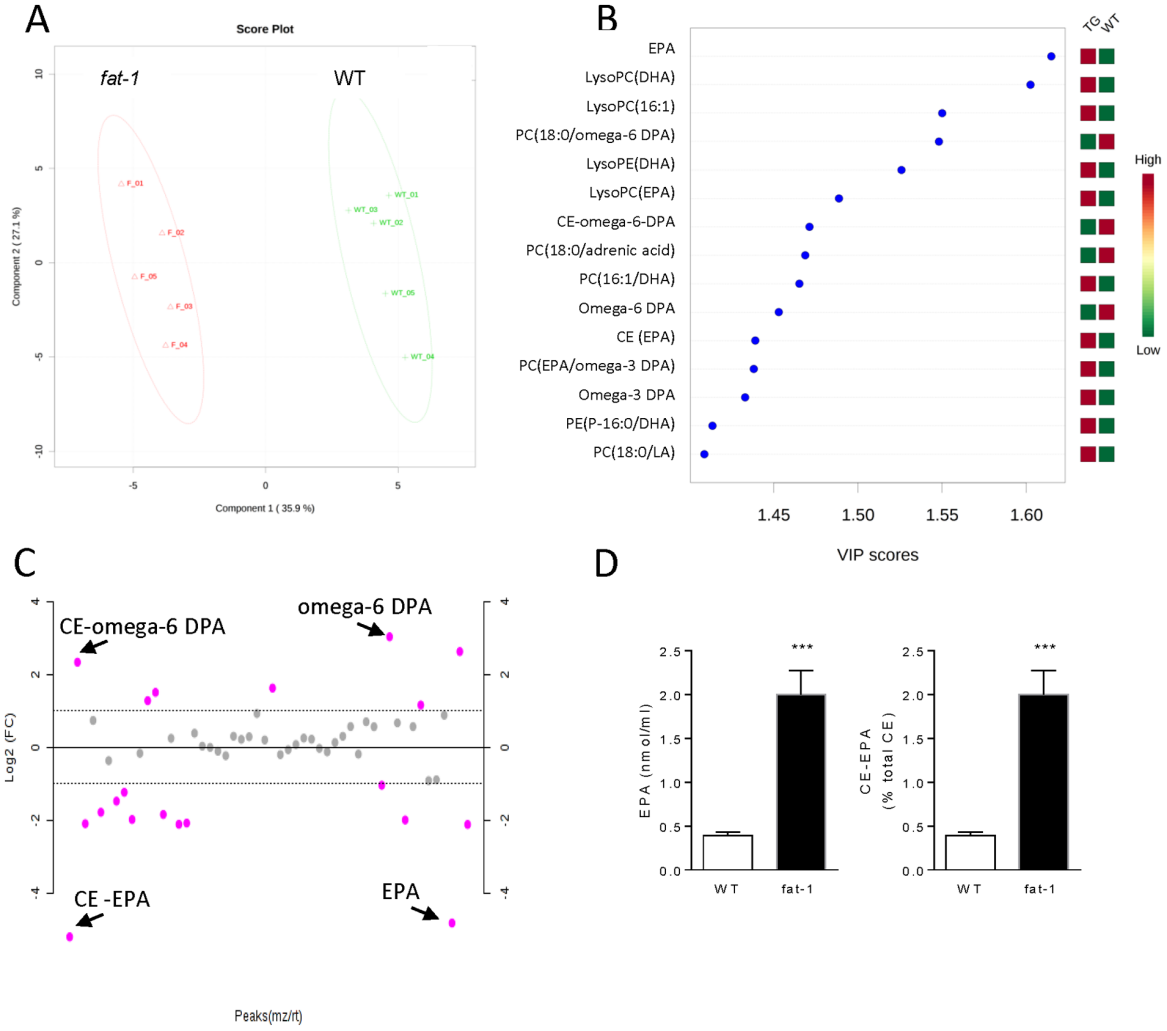
Untargeted lipidomic analysis. A, PLS-DA analysis showed a marked separation of plasma samples belonging to WT and *fat-1* mice, highlighting the features that contributed most to the variance between the two groups. B, Important features identified by PLS-DA. The colored boxes on the right indicate the relative concentrations of the corresponding metabolite in each group under study. Variable Importance in Projection (VIP) is a weighted sum of squares of the PLS loadings taking into account the amount of explained Y-variation in each dimension. C, Important features selected by fold-change analysis (relative to WT) with threshold 2. The red circles represent features above the threshold. Note the values are on log scale, so that both up-regulated and downregulated features can be plotted in a symmetrical way. D, Levels of EPA and percent composition of CE-EPA in WT and *fat-1* mice (n = 5, Student's t test; ***, p<0.001). The data present the mean ± SEM.

To identify the most abundant lipid alterations resulting from a balanced omega-6/omega-3 tissue ratio, we adopted an untargeted lipidomic approach. Using electrospray-mass spectrometry in both positive and negative ionization mode, we detected thousands of molecular features in plasma samples. Significant changes were identified using ANOVA (Table S3). Multivariate statistics, including PCA (Figure S1) and PLS-DA, highlighted the molecular features contributing most to the variance between the two groups of mice (Figure 3A and B). A fold-change analysis showed a marked increase in the levels of EPA and EPA-containing cholesteryl ester (CE-EPA) (Figure 3C and D), whereas a corresponding, significant decrease in the levels of omega-6 DPA and DPA-containing CE (CE-omega-6-DPA) in *fat-1* mice compared to WT littermates (Figure 3C). Slight but significant increases were also observed for unesterified DHA and CE-DHA, accompanied by a modest decrease for unesterified AA and CE-AA (Tables 1 and 2).

**Table 1:** Levels of unesterified fatty acids (nmol/ml) in WT and *fat-1* mice (n = 5).

Fatty acid species	WT	*fat-1*	p-value	FDR
18:3 n-3 (ALA)	24.33±1.49	24.49±1.96	0.9484737	0.63176
20:5 n-3 (EPA)	2.19±0.16	17.04±0.36	2.558E-10	0.001165
22:5 n-3 (omega-3 DPA)	0.96±0.09	3.39±0.19	2.473E-06	0.009725
22:6 n-3 (DHA)	22.92±1.68	27.10±0.91	0.0017493	0.81954
Total n-3	50.40±2.41	72.02±2.54	0.0002642	
18:2 n-6 (LA)	64.64±7.42	50.06±3.51	0.1135015	0.15112
20:3 n-6 (DGLA)	8.32±0.74	7.06±0.19	0.1382162	0.25896
20:4 n-6 (AA)	48.72±6.21	34.63±1.62	0.0596187	0.10569
22:4 n-6 (adrenic acid)	2.10±0.13	1.53±0.2	0.0440865	0.15112
22:5 n-6 (omega-6 DPA)	8.16±0.6	3.18±0.33	8.443E-05	0.009361
Total n-6	131.93±13.35	96.45±4.96	0.0374117	
Omega-6/omega-3 ratio	2.61±0.21	1.34±0.04	0.0003226	

P- values derived from Student's t test. FDR, false discovery rate.

**Table 2:** Composition (% of the total) of cholesteryl esters in WT and *fat-1* mice (n = 5).

CE species	WT	*fat-1*	p-value	FDR
18:3 n-3	1.17±0.1	1.16±0.03	0.8664	0.74976
20:5 n-3	0.39±0.04	2.08±0.27	0.0002	0.009725
22:5 n-3	1.00±0.13	1.36±0.11	0.039	0.008543
22:6 n-3	9.66±0.63	12.73±0.54	0.0054	0.58567
18:2 n-6	24.9±1.08	25.72±1.08	0.4847	0.86083
20:3 n-6	7.96±0.44	7.75±0.29	0.6161	0.63176
20:4 n-6	50.94±0.58	45.81±1.49	0.009	0.42803
22:5 n-6	0.82±0.07	0.21±0.04	0.0001	0.008543
18:1 n-9	3.18±0.6	3.18±0.27	0.9351	0.77652

P-values derived from Student's *t* test.

The plasma of *fat-1* mice exhibited a balanced omega-6/omega-3 ratio for unesterified PUFA, differing from the plasma of the WT littermates, for which the ratio shifted toward the omega-6 PUFAs (Table 1). Other significant differences between *fat-1* and WT mice include the levels of phosphatidylcholines (PCs) and phosphatidylethanolamines (PEs) (Figure 3B and Table S3). Most notably, we observed a marked increase in the levels of various lysophospholipids, including DHA-containing lyso PC and lyso PE, in *fat-1* mice (Figure 3B). Finally, the levels of various TG species containing omega-6 fatty acids, as well as cholesterol and cholesterol sulfate, were also decreased in *fat-1* mice (Table S3).

**Figure 4:**
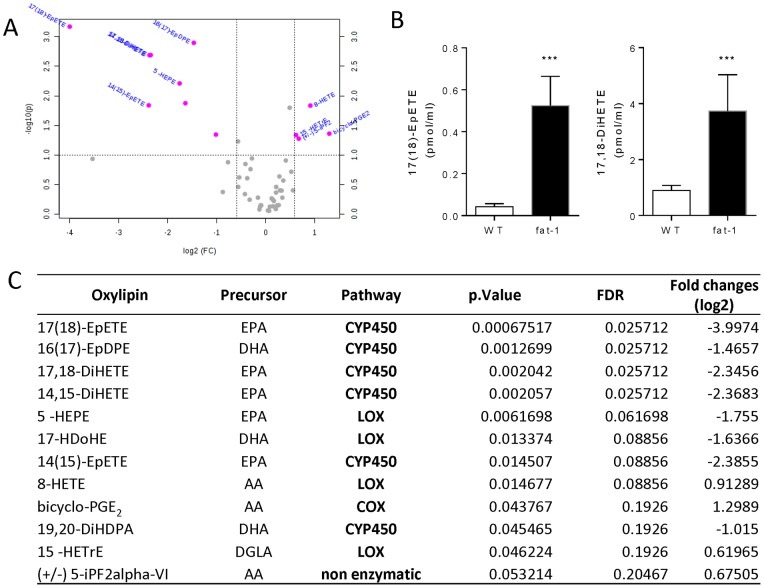
Targeted lipidomic analyses. A, Important features selected by volcano plot with fold change threshold (x) 2 and t-tests threshold (y) 0.1. The red circles represent features above the threshold. Note both fold changes (relative to WT) and p values are log transformed. The further its position away from the (0,0), the more significant the feature is. B, Levels of representative EPA-derived epoxide and diol species in WT and *fat-1* mice (n = 5, Student's t test; ***, p<0.001). The data present the mean ± SEM. C, Most important oxylipins features as determined by volcano plot; also reported: Student's t test p-value, false discovery rate (FDR) and fold-change.

PUFAs can be converted into hundreds of bioactive oxygenated metabolites (i.e., oxylipins) by means of enzymatic and nonenzymatic reactions. To measure the levels of such usually low-abundance metabolites, we used a multiplexed-targeted lipidomic approach (Figure 2). We measured the levels of more than 100 common oxylipins using tandem MS (multiple reaction monitoring). A volcano plot analysis using Student t-test p<0.01 and a fold change >2 as cut-off parameters highlighted a marked increase in the levels of EPA epoxides and diols in the plasma of *fat-1* versus WT mice, including 17(18)-epoxy-eicosatetraenoic acid (17(18)-EpETE) and 17,18-dihydroxy-eicosatetraenoic acid (17,18-DiHETE) (Figure 4B). Such differences were found to be significant after multiple comparison correction (Figure 4C and Table S1). Minor increases were also observed for other EPA- or DHA-derived metabolites (Figure 4C), whereas decreases were observed in the levels of DGLA- and AA-derived metabolites in *fat-1* mice (Figure 4C and Table 1).

**Figure 5:**
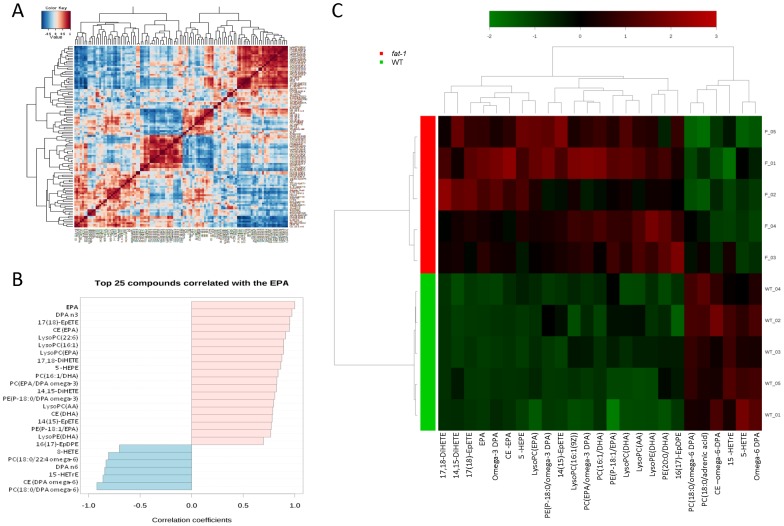
Lipidomic biosignature of *fat-1* mice. A, Correlation analysis was used to visualize the overall relationships between different features and (B) to identify which features are correlated with EPA. C, Clustering result shown as heatmap (distance measure using Pearson, and clustering algorithm using ward), providing an intuitive visualization of the characteristic lipidomic biosignature found in *fat-1* mice versus WT mice. Each colored cell on the map corresponds to a concentration value, with samples in rows and features/compounds in columns. Displayed are the top 25 lipids ranked by t-tests.

To determine the overall differences in the molecular phenotypes of *fat-1* and WT mice, we combined the results derived from both the untargeted and targeted analyses. Correlation analysis revealed a marked association of many features with EPA and EPA metabolites (Figures 5A and B). Thes lipidomic biosignature for *fat-1* mice was underscored by a significant molecular contrast between the two groups of mice (Figure 5C).

## Discussion

In this study, we compared the plasma lipidome of transgenic *fat-1* and WT mice after exposing the animals for six months to a diet high in omega-6 content. The *fat-1* mice are able to endogenously convert omega-6s into omega-3s, serving as a good model of a balanced omega-6/omega-3 ratio [3], [15], [16]. The design of this comparative study allowed an investigation of the effects of long-term omega-3 PUFA enrichment without the need for two different diets, thereby eliminating confounders derived from differences in calories, flavor, or nutrients. Our approach also avoided other confounders associated with individual genetic and environmental variability, which can be found in nutritional intervention studies involving human subjects [14], [22].

Previous studies have determined the total fatty acid composition of various tissues from *fat-1* mice using gas chromatography (GC), showing that the omega-6/omega-3 PUFA ratio can range from about 1 to 5 depending on the tissue, while wild type mice usually range from 20–50 (with the exception of the brain which is about 4) [3], [15], [16], [22]. Such studies, however, aimed to measure the total fatty acid content without detailing information on the levels of unesterified fatty acids, their product of oxygenation (oxylipins), and complex lipid species carrying PUFA, which may be particularly enriched in the plasma lipoproteins of fasted animals. By using two complementary analytical approaches, untargeted and multiplexed targeted lipidomic analyses, we were able to compare, in a more comprehensive fashion, the molecular phenotypes of the plasma from *fat-1* and WT mice. Indeed, our study provided information about the availability of unesterified fatty acids that might serve as precursors for the formation of oxylipins. Furthermore, our study described the rearrangement of fatty acyl chains in complex lipid structures, which might determine 1) the physicochemical properties of the membranes and lipoproteins containing such lipids and 2) the ability to transport PUFAs to various organs [15], [22].

The most obvious observation deriving from the untargeted lipidomic analysis is a rearrangement of the fatty acyl chains of CEs and phospholipids, which largely reflected the availability of omega-3 and omega-6 PUFAs. Such results are consistent with previous animal and human studies showing that blood lipid composition reflects the intake of PUFAs [14], [23]–[28]. We observed, however, specific alterations of PUFA-containing lipids that appear to be independent from the PUFA availability. In particular, we observed a marked increase for CE-EPA in the plasma of the *fat-1* mice, as compared to CE-DHA (Table 2). This observation generally aligns with previous reports showing a limited effect on CE-DHA concentration following supplementation with high doses of DHA in humans [29], [30]. Although some have speculated that a retroconversion of DHA to EPA could contribute to the limited biosynthesis of CE-DHA [31]–[37], this biosynthesis could also come about because of differential specificity of enzymes involved in the metabolism of PC species carrying DHA and EPA. Previous studies indeed showed that the CE composition is largely determined by the specificity of the enzyme lecithin-cholesterol acyl transferase (LCAT). The modest changes observed for CE-DHA may therefore arise because DHA is a poor substrate for LCAT, compared with EPA [26], [38]–[41]. Although the literature included relatively little evidence regarding the role of different cholesteryl esters, the observed increase in CE-EPA might affect the size or action of lipoproteins [42], [43].

Determining the plasma profile of unesterified fatty acids is normally problematic due to the direct influence of diet on plasma fatty acid content. Using a single diet for both WT and *fat-1* animals made it possible for plasma content analysis to be used as a reliable indicator of endogenous PUFA metabolism. Notably, the diet does not contain longer chain PUFAs (such as AA, EPA, and DHA) and any circulating lipids longer than 18 carbons must come from endogenous metabolism of shorter PUFAs. Our study highlighted a significant difference in the omega-6/omega-3 ratio between unesterified plasma lipids and other previously reported tissues [3], [15], [16]. In particular, our results showed a marked increase for unesterified EPA compared to unesterified DHA in the plasma of *fat-1* mice (Table 1), which could be explained, in part, by a retroconversion of DHA to EPA [31]–[37]. The concomitant elevation of the DHA-containing phospholipids, however, suggests that EPA might be a preferred substrate for the hydrolytic activity of phospholipase A_2_ (PLA_2_) in plasma, rather than DHA [44]. These observations point to a differential metabolism for EPA and DHA, which could explain the diverse physiological effects previously reported for these two omega-3 PUFAs [45]–[47]. On the other hand, DHA-containing phospholipids and lysophospholipids may play roles in the cellular membrane properties and in the transport of DHA to other tissues [40], [42]. Lysophospholipids containing DHA do, in fact, appear to be best suited as carriers of this essential fatty acid to eye and brain tissues, where it modulates the membrane fluidity of synaptic vesicles [48]–[50] and displays neuroprotective properties [51]. Notably, our untargeted analysis highlights a wide increase in other lysophospholipid species, which comports with a previous report showing that dietary supplementation with omega-3s can dynamically regulate plasma lysoPC [52].

The untargeted lipidomic analysis also showed a significant decrease in triacylglycerols and cholesterol. Such a decrease is in accordance with the fact that the *fat-1* phenotype is resistant to metabolic syndrome, obesity, and liver steatosis [53]–[56]. Significantly, the unexpected decrease in cholesterol-sulfate in the plasma of *fat-1* mice may also be linked with some of the protective effect of omega-3s. Cholesterol sulfate is present in lipoproteins, and it has been found in atherosclerotic lesions of the human aorta, where it plays a role in platelet adhesion, possibly determining the prothrombotic potential of atherosclerotic lesions [57]. Cholesterol sulfate is also found to be particularly enriched in DHA-rich cellular membranes, where it seems to modulate the lipid raft formation [58], [59]. One might reasonably speculate that the observed decrease in the circulating levels of cholesterol sulfate might indicate the effect of possible sequestration caused by the DHA-rich cellular membranes in *fat-1* mice. To establish the validity of such a hypothesis is beyond the scope of this paper.

**Figure 6:**
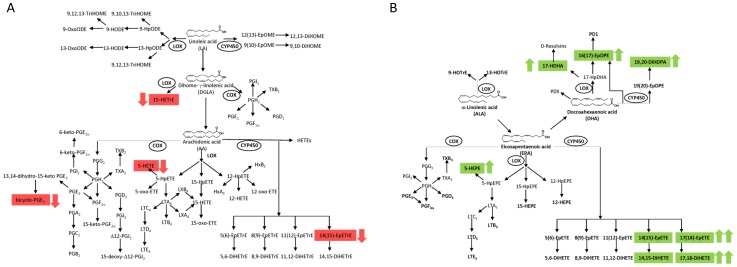
Pathway analysis. The activities of COX, LOX and CYP450 enzymes catalyze the formation of hundreds of oxylipins species with different biological activities starting from the omega-6 PUFAs precursors (*panel A*) and the omega-3 PUFAs (*panel B*). The *fat-1* mice had marked alterations in the CYP450 pathway and minor alterations in the LOX/COX pathways resulting in the increase of omega-3 oxylipins (green) and decrease of omega-6 oxylipins (red).

The multiplexed targeted lipidomics experiments highlighted the marked separation of the panel of oxylipins in the plasma of WT and *fat-1* mice. These lipid mediators are currently the focus of considerable interest, for they are also key messengers for cellular homeostasis, inflammation, platelet aggregation, and vascularization [11]–[13]. Oxylipins are produced via enzymatic or nonenzymatic oxygenation of both omega-6 and omega-3 PUFAs. Three major enzymatic pathways are involved in their generation: cyclooxygenase (COX), lipoxygenase (LOX) and cytochrome P450 (CYP) (Figures 6A and 6B). These pathways are important drug targets for various diseases. The ability to control such pathways with dietary interventions (i.e., omega-3 supplementation) could offset many of the side effects linked to pharmacological treatments. Our study indicated that the availability of unesterified omega-3 PUFA precursors correlated with the increase in the levels of the corresponding omega-3 oxylipins while decreasing the omega-6 oxylipins. Such results suggest that the higher levels of omega-3s compete with omega-6s for enzymatic and nonenzymatic activities to ultimately reduce the pro-inflammatory lipid mediators (HETEs, bicyclo-PGE_2_ and (+/−)5-iPF2alpha-VI) while increasing the levels of anti-inflammatory mediators (5-HEPE, 15-HETrE, and 17-HDoHE).

Notably, the most abundant oxylipins alterations in *fat-1* mice were related to the metabolism of the CYP450 pathway [37] (Figure 6). The CYP450 family of enzymes can produce epoxides from PUFAs, which are subsequently metabolized by the soluble epoxide hydrolase (sEH) to the corresponding vicinal diols, dihydroxyeicosatrienoic acids [60]. In human and animal studies, the CYP-dependent metabolite profiles were generally reflective of the PUFA composition [14], [37], [61]–[63], suggesting that most of the CYP-epoxygenases accept omega-3s and omega-6s as equally efficient substrates [64]. Recent evidence shows that DHA intake increases the levels of the EPA-derived vicinal diol 17,18-DiHETE metabolized in the CYP/sEH pathway in plasma from piglets, suggesting that DHA retroconversion to EPA may occur to some extent [37].

Although the physiologic properties of AA-derived metabolites of CYP have been studied extensively [65]–[67], the study of DHA and EPA-derived metabolites of CYP450 and their physiologic properties has only recently begun. Omega-3 CYP-metabolites have been described as possessing anti-inflammatory [68] and analgesic properties [69], as inhibitors of platelet aggregation [70], and as pulmonary, smooth-muscle relaxants [71]. It has been suggested that some of the beneficial effects of fish-oil-enhanced diets on cardiovascular function may be mediated by the levels of these metabolites [72]. Our results support the hypothesis that CYP-450-mediated omega-3 metabolism might represent a major physiological pathway underlying the reduced disease risk and health benefits observed across numerous *fat-1* mouse studies.

Finally, the integration of untargeted and targeted lipidomic results provided a detailed molecular signature for a balanced omega-6/omega-3 tissue ratio. Overall, EPA levels were the most remarkable molecular change observed in the plasma of *fat-1* mice. Although the use of the *fat-1* transgenic mouse model allowed us to eliminate confounding factors of the diet, further work would be needed to establish the validity of these molecular changes in humans.

In conclusion, this study demonstrates a protective lipidomic biosignature in *fat-1* mice that may contribute to the low risk of disease and health benefits associated with a balanced omega-6/omega-3 tissue ratio. Such a lipidomic biosignature could be used as a potential circulating biomarker for monitoring the “health status” or the efficacy of nutritional intervention with omega-3s in humans.

## Supporting Information

Figure S1 PCA plot have been applied to the ions that were found to have statistically significant alterations in *fat-1* mice compared to WT mice. The separation between clusters of the samples from *fat-1* transgenic mice (TG in red) and WT mice (in green) is indicative of the potential discriminating power of the statistically significant lipid identified.
(TIF)

Table S1 Nutritional and fatty acid composition of the 10% corn oil diet.
(DOCX)

Table S2 Levels of oxylipins expressed as mean concentration and SEM values (n = 5) for each group (*fat-1* and WT).
(DOCX)

Table S3 Tentative identification of the most significant lipid alterations obtained from the unbiased lipidomic analysis.
(DOCX)
