# Supplementary figures and images for: A Protective Lipidomic Biosignature Associated with a Balanced Omega-6/Omega-3 Ratio in fat-1 Transgenic Mice
Source: PLoS One. 2014 Apr 23;9(4):e96221. doi: 10.1371/journal.pone.0096221 (PMC3997567; doi:10.1371/journal.pone.0096221)

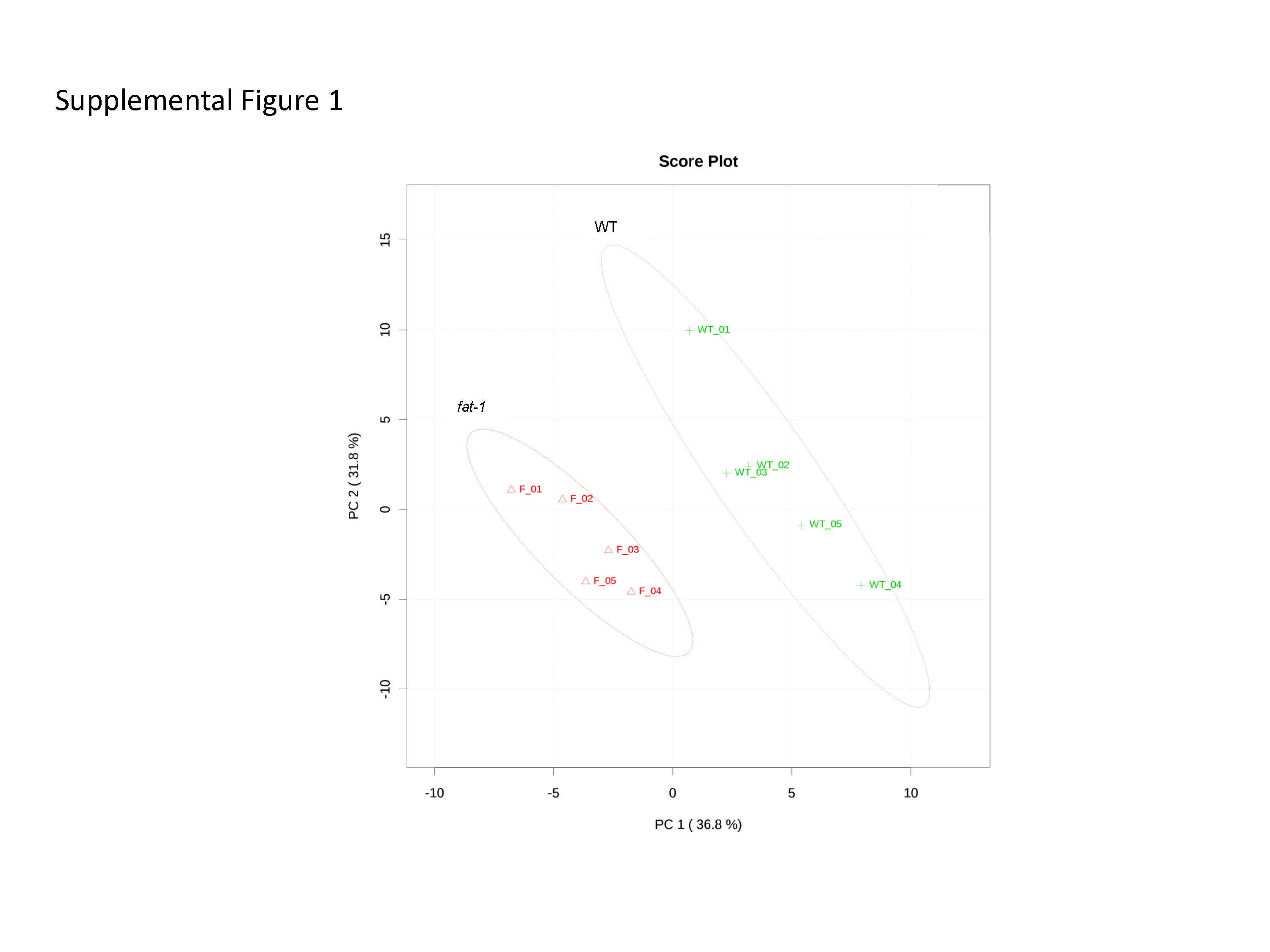

Supplement: Figure S1 — PCA plot have been applied to the ions that were found to have statistically significant alterations in fat-1 mice compared to WT mice. The separation between clusters of the samples from fat-1 transgenic mice (TG in red) and WT mice (in green) is indicative of the potential discriminating power of the statistically significant lipid identified. (TIF) [file pone.0096221.s001.tif]
