# Supplementary material for: A Protective Lipidomic Biosignature Associated with a Balanced Omega-6/Omega-3 Ratio in fat-1 Transgenic Mice
Source: PLoS One. 2014 Apr 23;9(4):e96221. doi: 10.1371/journal.pone.0096221 (PMC3997567; doi:10.1371/journal.pone.0096221)
Supplement: Table S1 — Nutritional and fatty acid composition of the 10% corn oil diet. (DOCX) [file pone.0096221.s002.docx]

**Table S1.** Nutritional facts and fatty acid composition of the 10% corn oil diet.

|  | Nutrition Facts |  | Fatty acid Composition  (% total fatty acids) | |
| --- | --- | --- | --- | --- |
| PRO kcal% | 16.8 |  | C8:0 | 0.1 |
| CHO kcal% | 51.4 |  | C10:0 | 0.27 |
| FAT kcal% | 31.8 |  | C12:0 | 0.7 |
| PRO (g) | 21.3 |  | C16:0 | 14.75 |
| Casein | 21 |  | C16:1 | 0.18 |
| DL-Methionine | 0.3 |  | C18:0 | 2.36 |
| CHO (g) | 59 |  | C18:1 | 28.22 |
| Corn starch | 25.4 |  | C18:2 | 52.32 |
| Sucrose | 33.6 |  | C18:3 n-3 | 1.1 |
| FAT (g) | 10 |  | SFA | 18.19 |
| corn oil | 10 |  | Total | 100 |
| Cellulose | 5 |  |  |  |
| Mineral Mix S10001 | 3.5 |  | MUFA | 28.4 |
| Vitamine Mix V10001 | 1 |  | PUFA | 53.42 |
| Choline Bitartrate | 0.2 |  | n-6 PUFA | 52.32 |
| Total | 100 |  | n-3 PUFA | 1.1 |
| Kcal/gm | 4.11 |  | n-6/n-3 | 47.56 |
