# Supplementary material for: A Protective Lipidomic Biosignature Associated with a Balanced Omega-6/Omega-3 Ratio in fat-1 Transgenic Mice
Source: PLoS One. 2014 Apr 23;9(4):e96221. doi: 10.1371/journal.pone.0096221 (PMC3997567; doi:10.1371/journal.pone.0096221)
Supplement: Table S2 — Levels of oxylipins expressed as mean concentration and SEM values (n = 5) for each group (fat-1 and WT). (DOCX) [file pone.0096221.s003.docx]

**Table S2.**  Levels of oxylipins expressed as mean concentration and SEM values (n = 5) for each group (*fat-1* and WT). Oxylipins are ranked according to their Student’s *t* test p values. Retention times (RT), monitored transitions (M1>M2) and internal standards (ISTD) used for quantification are also reported.

| **Oxylipin** | **WT** | ***fat-1*** | **p-value** | **FDR** | **M1** | **M2** | **RT** | **ISTD** | **Precursor** | **Class** | **Pathway** |
| --- | --- | --- | --- | --- | --- | --- | --- | --- | --- | --- | --- |
| 17(18)-EpETE | 0.04 ± 0.01 | 0.52 ± 0.11 | 0.0006752 | 0.025712 | 317.2 | 259.2 | 5.16 | (d11) 14,15-DiHETrE | EPA | epoxide | CYP450 |
| 16(17)-EpDPE | 0.06 ± 0.01 | 0.13 ± 0.03 | 0.0012699 | 0.034283 | 343.2 | 233.2 | 5.62 | (d11) 14,15-DiHETrE | DHA | epoxide | LOX/CYP450 |
| 17,18-DiHETE | 0.9 ± 0.08 | 3.73 ± 0.57 | 0.002042 | 0.034283 | 335.2 | 247.2 | 4.34 | (d11) 14,15-DiHETrE | EPA | diol | CYP450 |
| 14,15-DiHETE | 0.06 ± 0.02 | 0.26 ± 0.06 | 0.002057 | 0.034283 | 335.2 | 207.1 | 4.46 | (d11) 14,15-DiHETrE | EPA | diol | CYP450 |
| 5 -HEPE | 0.5 ± 0.08 | 1.39 ± 0.34 | 0.0061698 | 0.077123 | 317.2 | 115.1 | 5.51 | (d8) 5(S)-HETE | EPA | alcohol | LOX |
| 17-HDoHE | 0.28 ± 0.11 | 0.84 ± 0.23 | 0.013374 | 0.08856 | 343.2 | 281.3 | 5.67 | (d8) 5(S)-HETE | DHA | alcohol | LOX |
| 14(15)-EpETE | 0.01 ± 0 | 0.05 ± 0.01 | 0.014507 | 0.08856 | 317.2 | 207.1 | 5.74 | (d11) 14,15-DiHETrE | EPA | epoxide | CYP450 |
| 8-HETE | 27.5 ± 5.23 | 12.67 ± 2.88 | 0.014677 | 0.08856 | 319.2 | 155.1 | 5.85 | (d8) 5(S)-HETE | AA | alcohol | LOX |
| 5-HETE | 12.17 ± 0.88 | 7.24 ± 1.09 | 0.015941 | 0.08856 | 319.2 | 115.1 | 6.00 | (d8) 5(S)-HETE | AA | alcohol | LOX |
| bicyclo-PGE2 | 0.22 ± 0.03 | 0.08 ± 0.06 | 0.043767 | 0.1926 | 333.2 | 113.2 | 4.25 | (d4) PGE2 | AA | prostanoid | COX |
| 19,20-DiHDPA | 1.63 ± 0.28 | 2.7 ± 0.3 | 0.045465 | 0.1926 | 361.2 | 273.3 | 4.79 | (d11) 14,15-DiHETrE | DHA | diol | CYP450 |
| 15 -HETrE | 6.65 ± 0.82 | 3.82 ± 0.48 | 0.046224 | 0.1926 | 321.2 | 221.2 | 5.88 | (d8) 5(S)-HETE | DGLA | alcohol | LOX |
| (+/-) 5-iPF2alpha-VI | 3.16 ± 0.57 | 1.58 ± 0.34 | 0.053214 | 0.20467 | 353.2 | 115.1 | 3.04 | (d4) PGF2α | AA | isoprostane | non enzymatic |
| 9-HODE | 110.94 ± 21.93 | 142.51 ± 26.64 | 0.059324 | 0.21187 | 295.2 | 171.1 | 5.56 | (d4) 9(S)-HODE | LA | alcohol | LOX |
| PGE2 | 1.17 ± 0.15 | 1.2 ± 0.11 | 0.11511 | 0.36626 | 351.2 | 271.2 | 3.2 | (d4) PGE2 | AA | prostanoid | COX |
| 12 -HEPE | 13.4 ± 3.69 | 142.93 ± 88 | 0.1172 | 0.36626 | 317.2 | 179.1 | 5.35 | (d8) 12(S)-HETE | EPA | alcohol | LOX |
| 15-KETE | 6.45 ± 0.67 | 4.05 ± 0.64 | 0.12475 | 0.36691 | 317.2 | 113.2 | 5.72 | (d8) 5(S)-HETE | AA | ketone | LOX |
| 15-HEPE | 1.05 ± 0.12 | 1.65 ± 0.52 | 0.1335 | 0.37084 | 317.2 | 219.2 | 5.25 | (d8) 5(S)-HETE | EPA | alcohol | LOX |
| 12,13-DiHOME | 86.93 ± 12.18 | 96.55 ± 17.23 | 0.14323 | 0.37692 | 313.2 | 183.2 | 4.58 | (d4) 12,13-DiHOME | LA | diol | CYP450 |
| 9,10-DiHOME | 65.74 ± 9.32 | 67.8 ± 8.67 | 0.17494 | 0.43736 | 313.2 | 201.1 | 4.71 | (d4) 9,10-DiHOME | LA | diol | CYP450 |
| 8(9)-EpETrE | 0.7 ± 0.17 | 0.39 ± 0.05 | 0.1936 | 0.46095 | 319.2 | 155.1 | 6.33 | (d11) 14,15-DiHETrE | AA | epoxide | CYP450 |
| 14,15-DiHETrE | 5.77 ± 1.03 | 4 ± 0.46 | 0.23266 | 0.51852 | 337.2 | 207.2 | 4.8 | (d11) 14,15-DiHETrE | AA | diol | CYP450 |
| 15-keto PGF1alpha | 0.86 ± 0.14 | 1.15 ± 0.25 | 0.24149 | 0.51852 | 353.2 | 221.1 | 3.37 | (d4) 6-keto PGF1α | DGLA | prostanoid | COX |
| 13-HODE | 310.55 ± 71.55 | 348.75 ± 70.21 | 0.24889 | 0.51852 | 295.2 | 195.2 | 5.50 | (d4) 9(S)-HODE | LA | alcohol | LOX |
| 14(15)-EpETrE | 0.18 ± 0.04 | 0.12 ± 0.01 | 0.27301 | 0.54603 | 319.2 | 219.2 | 6.11 | (d11) 14,15-DiHETrE | AA | epoxide | CYP450 |
| LTB5 | 0.22 ± 0.06 | 0.25 ± 0.05 | 0.34962 | 0.64846 | 333.2 | 195.1 | 4.03 | (d4) LTB4 | EPA | leukotrien | LOX |
| 11,12-DiHETrE | 2.57 ± 0.45 | 1.86 ± 0.26 | 0.35017 | 0.64846 | 337.2 | 167.2 | 4.98 | (d11) 14,15-DiHETrE | AA | diol | CYP450 |
| 5-KETE | 2.04 ± 0.38 | 1.4 ± 0.31 | 0.40031 | 0.6734 | 317.2 | 203.2 | 6.26 | (d8) 5(S)-HETE | AA | ketone | LOX |
| 20-HETE | 4.11 ± 1.55 | 2.41 ± 0.57 | 0.40046 | 0.6734 | 319.2 | 289.2 | 5.25 | d6-20-HETE | AA | alcohol | CYP450 |
| TXB2 | 6.18 ± 1 | 4.52 ± 1.12 | 0.40404 | 0.6734 | 369.2 | 169.1 | 2.88 | (d4) TXB2 | AA | thromboxane | COX |
| 12-HHTrE | 3.2 ± 0.42 | 5.4 ± 0.68 | 0.42639 | 0.68138 | 279.2 | 179.2 | 4.84 | (d8) 12(S)-HETE | AA | alcohol | COX |
| 11-HETE | 22.8 ± 3.05 | 16.87 ± 1.11 | 0.43609 | 0.68138 | 319.2 | 167.1 | 5.74 | (d8) 12(S)-HETE | AA | alcohol | COX |
| PGD2 | 0.48 ± 0.13 | 0.59 ± 0.19 | 0.46248 | 0.70073 | 351.2 | 271.2 | 3.32 | (d4) PGD2 | AA | prostanoid | COX |
| 9(10)-EpOME | 86.99 ± 14.17 | 80.55 ± 9.32 | 0.53078 | 0.75739 | 295.2 | 171.2 | 6.15 | (d4) 9,10-DiHOME | LA | epoxide | CYP450 |
| 19(20)-EpDPE | 0.44 ± 0.06 | 0.3 ± 0.08 | 0.53123 | 0.75739 | 343.2 | 281.3 | 6.09 | (d11) 14,15-DiHETrE | DHA | epoxide | CYP450 |
| 5(6)-EpETrE | 0.48 ± 0.06 | 0.36 ± 0.05 | 0.54796 | 0.75739 | 319.2 | 191.2 | 6.42 | (d11) 14,15-DiHETrE | AA | epoxide | CYP450 |
| 13-HpODE | 1.76 ± 0.23 | 2.03 ± 0.61 | 0.57509 | 0.75739 | 311.2 | 113.2 | 5.63 | (d4) 9(S)-HODE | LA | hydroxyperoxide | LOX |
| 9-KODE | 39.6 ± 8.74 | 29.63 ± 4.02 | 0.57562 | 0.75739 | 293.2 | 185.2 | 5.77 | (d4) 9(S)-HODE | LA | ketone | LOX |
| 11(12)-EpETrE | 0.65 ± 0.12 | 0.47 ± 0.07 | 0.60489 | 0.7755 | 319.2 | 167.1 | 6.27 | (d11) 14,15-DiHETrE | AA | epoxide | CYP450 |
| 12 -HpETE | 2.45 ± 0.9 | 1.79 ± 0.83 | 0.71044 | 0.82309 | 335.2 | 273.3 | 5.34 | (d8) 12(S)-HETE | AA | hydroxyperoxide | LOX |
| 13-KODE | 48.6 ± 8.12 | 43.17 ± 7.41 | 0.71482 | 0.82309 | 293.2 | 113.1 | 5.64 | (d4) 9(S)-HODE | LA | ketone | LOX |
| 12(13)-EpOME | 40.89 ± 6.34 | 36.18 ± 3.86 | 0.71769 | 0.82309 | 295.2 | 195.2 | 6.09 | (d4) 12,13-DiHOME | LA | epoxide | CYP450 |
| 15-deoxy-delta 12,14-PGD2 | 1.29 ± 0.22 | 0.94 ± 0.32 | 0.71927 | 0.82309 | 333.2 | 271.2 | 4.49 | (d4) 15-deoxy-Δ12,14-PGJ2 | AA | prostanoid | COX |
| 15-HETE | 6.37 ± 0.42 | 5.3 ± 1.88 | 0.74392 | 0.82309 | 319.2 | 219.2 | 5.62 | (d8) 5(S)-HETE | AA | alcohol | LOX |
| 5 ,6 -DiHETE | 0.3 ± 0.06 | 0.28 ± 0.04 | 0.75554 | 0.82309 | 335.2 | 115.1 | 5.35 | (d4) LTB4 | AA | diol | CYP450 |
| PGF2alpha | 3.5 ± 0.39 | 2.83 ± 0.44 | 0.75724 | 0.82309 | 353.2 | 193.2 | 3.14 | (d4) PGF2α | AA | prostanoid | COX |
| 12-HETE | 1559.94 ± 596.24 | 1215.38 ± 747.06 | 0.82609 | 0.87676 | 319.2 | 179.2 | 5.81 | (d8) 12(S)-HETE | AA | alcohol | LOX |
| 9-HpODE | 3.58 ± 1.08 | 3.36 ± 1.11 | 0.84169 | 0.87676 | 311.2 | 185.2 | 5.68 | (d4) 9(S)-HODE | LA | hydroxyperoxide | LOX |
| LTE4 | 0.26 ± 0.04 | 0.21 ± 0.02 | 0.87509 | 0.88938 | 438.2 | 333.2 | 4.13 | (d3) LTE4 | AA | leukotrien | LOX |
| 13,14-dihydro-15-keto PGD2 | 0.57 ± 0.13 | 0.45 ± 0.14 | 0.88938 | 0.88938 | 351.2 | 175.2 | 3.77 | (d4) PGD2 | AA | prostanoid | COX |
| 10(S),17(S)-DiHDoHE | ND | ND | N/A | N/A | 359.2 | 153.2 | 4.34 | (d8) 12(S)-HETE | DHA | protectin | LOX |
| 11-trans LTD4 | ND | ND | N/A | N/A | 495.2 | 177.1 | 4.05 | (d3) LTE4 | AA | leukotrien | LOX |
| 11-trans LTE4 | ND | ND | N/A | N/A | 438.2 | 333.2 | 4.26 | (d3) LTE4 | AA | leukotrien | LOX |
| 11β-13,14-dihydro-15-keto PGF2α | ND | ND | N/A | N/A | 353.2 | 113.2 | 3.35 | (d4) PGF2α | AA | prostanoid | COX |
| 11β-PGE2 | ND | ND | N/A | N/A | 351.2 | 271.2 | 3.25 | (d4) PGE2 | AA | prostanoid | COX |
| 11β-PGF2α | ND | ND | N/A | N/A | 353.2 | 193.2 | 2.93 | (d4) PGF2α | AA | prostanoid | COX |
| 12,13-DiHODE | ND | ND | N/A | N/A | 311.2 | 293 | 4.23 | (d4) 9,10-DiHOME | ALA | diol | CYP450 |
| 12-KETE | ND | ND | N/A | N/A | 317.2 | 273.3 | 6.25 | (d8) 12(S)-HETE | AA | ketone | LOX |
| 12S-HHTrE | ND | ND | N/A | N/A | 279.2 | 179.2 | 4.84 | (d8) 12(S)-HETE | AA | alcohol | COX |
| 13,14-dihydro PGF2α | ND | ND | N/A | N/A | 355.2 | 275.2 | 3.39 | (d4) PGF2α | AA | prostanoid | COX |
| 13,14-dihydro-15-keto PGD1 | ND | ND | N/A | N/A | 353.2 | 209.1 | 3.91 | (d4) PGD2 | AA | prostanoid | COX |
| 13,14-dihydro-15-keto PGE2 | ND | ND | N/A | N/A | 351.2 | 175.2 | 3.54 | (d4) PGE2 | AA | prostanoid | COX |
| 13,14-dihydro-15-keto PGF1α | ND | ND | N/A | N/A | 355.2 | 193.2 | 3.72 | (d4) PGF2α | AA | prostanoid | COX |
| 13,14-dihydro-15-keto PGF2α | ND | ND | N/A | N/A | 353.2 | 183.1 | 3.56 | (d4) PGF2α | AA | prostanoid | COX |
| 14,15-LTE4 | ND | ND | N/A | N/A | 438.2 | 333.2 | 3.78 | (d3) LTE4 | AA | leukotrien | LOX |
| 15(S)-HETrE | ND | ND | N/A | N/A | 321.2 | 221.2 | 5.88 | (d8) 5(S)-HETE | DGLA | alcohol | LOX |
| 15-deoxy-Δ12,14-PGD2 | ND | ND | N/A | N/A | 333.2 | 271.2 | 4.49 | (d4) 15-deoxy-Δ12,14-PGJ2 | AA | prostanoid | COX |
| 15-HpETE | ND | ND | N/A | N/A | 335.2 | 113.1 | 5.71 | (d8) 5(S)-HETE | AA | hydroxyperoxide | LOX |
| 15-keto PGF2α | ND | ND | N/A | N/A | 351.2 | 219.1 | 3.28 | (d4) PGF2α | AA | prostanoid | COX |
| 1α,1b-dihomo PGF2α | ND | ND | N/A | N/A | 381.3 | 337.2 | 3.77 | (d4) PGF2α | ADA | prostanoid | COX |
| 2,3-dinor-11b PGF2α | ND | ND | N/A | N/A | 325.2 | 145.1 | 2.27 | (d4) PGF2α | AA | prostanoid | COX |
| 20-carboxy LTB4 | ND | ND | N/A | N/A | 365.2 | 347.2 | 2.35 | (d4) LTB4 | AA | leukotrien | LOX |
| 20-hydroxy LTB4 | ND | ND | N/A | N/A | 351.2 | 195.1 | 2.46 | (d4) LTB4 | AA | leukotrien | LOX |
| 20-hydroxy PGE2 | ND | ND | N/A | N/A | 367.2 | 287.2 | 1.01 | (d4) PGE2 | AA | prostanoid | COX |
| 5(S),14(R)-Lipoxin B4 | ND | ND | N/A | N/A | 351.2 | 221.2 | 3.29 | (d4) LTB4 | AA | lipoxin | LOX |
| 5(S),15(S)-DiHETE | ND | ND | N/A | N/A | 335.2 | 115.2 | 4.37 | (d4) LTB4 | AA | diol | CYP450 |
| 5(S),6(R)-Lipoxin A4 | ND | ND | N/A | N/A | 351.2 | 115.1 | 3.58 | (d4) LTB4 | AA | lipoxin | LOX |
| 5(S),6(S)-Lipoxin A4 | ND | ND | N/A | N/A | 351.2 | 115.1 | 3.68 | (d4) LTB4 | AA | lipoxin | LOX |
| 5(S)-HpETE | ND | ND | N/A | N/A | 335.2 | 203.2 | 6.11 | (d8) 5(S)-HETE | AA | hydroxyperoxide | LOX |
| 5,6-DiHETrE | ND | ND | N/A | N/A | 337.2 | 145.1 | 4.99 | (d11) 14,15-DiHETrE | AA | diol | CYP450 |
| 6-keto PGE1 | ND | ND | N/A | N/A | 367.2 | 143.1 | 2.37 | (d4) PGE2 | AA | prostanoid | COX |
| 6-keto PGF1α | ND | ND | N/A | N/A | 369.2 | 163.1 | 2.27 | (d4) 6-keto PGF1α | AA | prostanoid | COX |
| 6-trans-LTB4 | ND | ND | N/A | N/A | 335.2 | 195.1 | 4.4 | (d4) LTB4 | AA | leukotrien | LOX |
| 8(S),15(S)-DiHETE | ND | ND | N/A | N/A | 335.2 | 235.2 | 4.23 | (d4) LTB4 | AA | diol | CYP450 |
| 8,9-DiHETrE | ND | ND | N/A | N/A | 337.2 | 127 | 5.35 | (d11) 14,15-DiHETrE | AA | diol | CYP450 |
| 8-iso PGF2α | ND | ND | N/A | N/A | 353.2 | 193.2 | 2.87 | (d4) PGF2α | AA | isoprostane | non enzymatic |
| 9,10,13-TriHOME | ND | ND | N/A | N/A | 329.2 | 171.1 | 3.12 | (d4) 9(S)-HODE | LA | triol | LOX |
| 9,12,13-TriHOME | ND | ND | N/A | N/A | 329.2 | 211.2 | 3.07 | (d4) 9(S)-HODE | LA | triol | LOX |
| 9-HETE | ND | ND | N/A | N/A | 319.2 | 167.1 | 5.91 | (d8) 12(S)-HETE | AA | alcohol | non enzymatic |
| 9-HOTrE | ND | ND | N/A | N/A | 293.2 | 171.1 | 5.07 | (d4) 9(S)-HODE | ALA | alcohol | LOX |
| Hepoxilin A3 | ND | ND | N/A | N/A | 335.2 | 273.2 | 4.5 | (d8) 12(S)-HETE | AA | hepoxilin | LOX |
| LTB4 | ND | ND | N/A | N/A | 335.2 | 195.1 | 4.5 | (d4) LTB4 | AA | leukotrien | LOX |
| LTD4 | ND | ND | N/A | N/A | 495.2 | 177.1 | 3.9 | (d3) LTE4 | AA | leukotrien | LOX |
| PD1 | ND | ND | N/A | N/A | 359.2 | 206 | 4.34 | (d8) 12(S)-HETE | DHA | protectin | LOX |
| PGA2 | ND | ND | N/A | N/A | 333.2 | 271.2 | 3.91 | (d4) PGE2 | AA | prostanoid | COX |
| PGD1 | ND | ND | N/A | N/A | 353.2 | 273.2 | 3.32 | (d4) PGD2 | DGLA | prostanoid | COX |
| PGD3 | ND | ND | N/A | N/A | 349.2 | 269.2 | 2.92 | (d4) PGD2 | EPA | prostanoid | COX |
| PGE1 | ND | ND | N/A | N/A | 353.2 | 273.2 | 3.29 | (d4) PGE2 | DGLA | prostanoid | COX |
| PGE3 | ND | ND | N/A | N/A | 349.2 | 269.2 | 2.83 | (d4) PGE2 | EPA | prostanoid | COX |
| PGF1α | ND | ND | N/A | N/A | 355.2 | 293.2 | 3.14 | (d4) PGF2α | DGLA | prostanoid | COX |
| PGF2α | ND | ND | N/A | N/A | 353.2 | 193.2 | 3.14 | (d4) PGF2α | AA | prostanoid | COX |
| PGF3α | ND | ND | N/A | N/A | 351.2 | 193.2 | 2.75 | (d4) PGF2α | EPA | prostanoid | COX |
| PGJ2 | ND | ND | N/A | N/A | 333.2 | 233.1 | 3.97 | (d4) PGD2 | AA | prostanoid | COX |
| PGK2 | ND | ND | N/A | N/A | 349.2 | 205.1 | 3.28 | (d4) PGE2 | AA | prostanoid | COX |
| Resolvin D1 | ND | ND | N/A | N/A | 375.2 | 141 | 3.90 | (d11) 14,15-DiHETrE | DHA | resolvin | LOX |
| Resolvin E1 | ND | ND | N/A | N/A | 349.2 | 195 | 3.90 | (d11) 14,15-DiHETrE | EPA | resolvin | LOX |
| Tetranor-PGEM | ND | ND | N/A | N/A | 327.1 | 309.2 | 0.53 | (d4) PGE2 | AA | prostanoid | COX |
| Tetranor-PGFM | ND | ND | N/A | N/A | 329.2 | 311.2 | 0.48 | (d4) PGF2α | AA | prostanoid | COX |
| TXB1 | ND | ND | N/A | N/A | 371.2 | 171.1 | 2.79 | (d4) TXB2 | DGLA | thromboxane | COX |
| TXB3 | ND | ND | N/A | N/A | 367.2 | 169.1 | 2.48 | (d4) TXB2 | EPA | thromboxane | COX |
| Δ12-PGJ2 | ND | ND | N/A | N/A | 333.2 | 233.1 | 3.97 | (d4) 15-deoxy-Δ12,14-PGJ2 | AA | prostanoid | COX |
| Δ17-6-keto PGF1α | ND | ND | N/A | N/A | 367.2 | 163.1 | 1.76 | (d4) 6-keto PGF1α | AA | prostanoid | COX |
| **(d11) 14,15-DiHETrE** | **internal standard** | |  |  | **348.3** | **207.1** | **4.77** |  |  |  |  |
| **(d3) LTE4** | **internal standard** | |  |  | **441.2** | **336.2** | **4.12** |  |  |  |  |
| **(d4) 15-deoxy-Δ12,14-PGJ2** | **internal standard** | |  |  | **319.2** | **275.3** | **5.2** |  |  |  |  |
| **(d4) 6-keto PGF1α** | **internal standard** | |  |  | **373.2** | **167.2** | **2.28** |  |  |  |  |
| **(d4) 9(S)-HODE** | **internal standard** | |  |  | **299.2** | **172.1** | **5.53** |  |  |  |  |
| **(d4) LTB4** | **internal standard** | |  |  | **339.2** | **197.1** | **4.48** |  |  |  |  |
| **(d4) PGD2** | **internal standard** | |  |  | **355.2** | **275.2** | **3.31** |  |  |  |  |
| **(d4) PGE2** | **internal standard** | |  |  | **355.2** | **275.2** | **3.19** |  |  |  |  |
| **(d4) PGF2α** | **internal standard** | |  |  | **357.3** | **197.2** | **3.12** |  |  |  |  |
| **(d4) TXB2** | **internal standard** | |  |  | **373.2** | **173.1** | **2.86** |  |  |  |  |
| **(d4)(±)12,13-DiHOME** | **internal standard** | |  |  | **317.3** | **185.2** | **4.56** |  |  |  |  |
| **(d4)-(±)9,10-DiHOME** | **internal standard** | |  |  | **317.3** | **203.2** | **4.69** |  |  |  |  |
| **(d6) 20-HETE** | **internal standard** | |  |  | **325.3** | **279.2** | **5.24** |  |  |  |  |
| **(d8) 12(S)-HETE** | **internal standard** | |  |  | **327.3** | **184.2** | **5.78** |  |  |  |  |
| **(d8) 5(S)-HETE** | **internal standard** | |  |  | **327.3** | **116.1** | **5.97** |  |  |  |  |
