# Supplementary material for: A Protective Lipidomic Biosignature Associated with a Balanced Omega-6/Omega-3 Ratio in fat-1 Transgenic Mice
Source: PLoS One. 2014 Apr 23;9(4):e96221. doi: 10.1371/journal.pone.0096221 (PMC3997567; doi:10.1371/journal.pone.0096221)
Supplement: Table S3 — Tentative identification of the most significant lipid alterations obtained from the unbiased lipidomic analysis. (DOCX) [file pone.0096221.s004.docx]

**Table S3.** Tentative identification of the most significant lipid alterations obtained from the unbiased lipidomic analysis. Lipids were ranked according to their ANOVA p values. False Discovery Rate (FDR), the Variable Importance in Projection (VIP) derived from the PLS-DA analysis and the fold change are also reported.

| **Lipid species** | **HMDB ID** | **p value** | **FDR** | **VIP** | **Fold change** | **Highest Mean** |
| --- | --- | --- | --- | --- | --- | --- |
| CE(20:5) | HMDB06731 | 0.000000279 | 0.009725 | 1.4389 | 0.18791 | TG |
| EPA | HMDB01999 | 0.0000029 | 0.001165 | 1.6148 | 0.14286 | TG |
| Omega-6 DPA | HMDB01976 | 0.000110734 | 0.009361 | 1.4529 | 2.8566 | WT |
| LysoPE(22:6) | HMDB11496 | 0.00036217 | 0.004769 | 1.5258 | 0.25095 | TG |
| TG(18:1/22:5/20:4[iso6] | HMDB50150 | 0.000526002 | 0.041986 | 1.2714 | 3.0775 | WT |
| CE(22:5) | HMDB10374 | 0.000810744 | 0.008543 | 1.4711 | 4.9839 | WT |
| PC(18:0/22:5 | HMDB08055 | 0.000992025 | 0.003467 | 1.548 | 2.7276 | WT |
| LysoPC(20:4) | HMDB10395 | 0.0013616 | 0.02908 | 1.3176 | 0.75374 | WT |
| PE(P-18:0/22:5) | HMDB11393 | 0.002428108 | 0.027596 | 1.3289 | 0.1188 | TG |
| PC(18:0/22:4) | HMDB08054 | 0.002636071 | 0.008543 | 1.4686 | 2.3604 | WT |
| TG(16:1/20:4/22:5[iso6] | HMDB48845 | 0.002856092 | 0.16144 | 0.98611 | 1.8347 | WT |
| LysoPC(16:1) | HMDB10383 | 0.003976027 | 0.003467 | 1.55 | 0.30545 | TG |
| PE(P-16:0/22:6) | HMDB05780 | 0.004185123 | 0.011763 | 1.4135 | 0.55565 | TG |
| DHA | HMDB02183 | 0.004214478 | 0.81954 | 0.19551 | 0.94865 | TG |
| PC(18:0/20:5) | HMDB08050 | 0.00436685 | 0.29288 | 0.82142 | 1.1645 | WT |
| TG(16:0/22:5/20:4) [iso6] | HMDB44462 | 0.008746305 | 0.60027 | 0.47684 | 1.2052 | WT |
| PE(18:0/22:5) | HMDB09010 | 0.010859274 | 0.18297 | 0.95198 | 2.3682 | WT |
| PC(18:1/20:4) | HMDB08114 | 0.011315859 | 0.3007 | 0.80582 | 1.1603 | WT |
| PC(16:1/22:6) | HMDB08023 | 0.011654171 | 0.008543 | 1.4651 | 0.24653 | TG |
| PC(P-16:0/20:4) | HMDB11221 | 0.012470206 | 0.98824 | 0.027884 | 0.99274 | WT |
| PC(18:0/18:2) | HMDB08039 | 0.013342351 | 0.011763 | 1.4087 | 0.88583 | WT |
| LysoPC(20:5) | HMDB10397 | 0.014931996 | 0.008493 | 1.4887 | 0.37154 | TG |
| TG(18:0/18:2/22:5)[iso6] | HMDB45092 | 0.015227154 | 0.46546 | 0.63452 | 1.4856 | WT |
| TG(16:0/14:0/18:2)[iso6] | HMDB10415 | 0.015719484 | 0.60027 | 0.49292 | 0.90064 | WT |
| PC(20:5/22:5) | HMDB08517 | 0.016710069 | 0.009725 | 1.4381 | 0.24442 | TG |
| AA | HMDB01043 | 0.018394383 | 0.10569 | 1.1013 | 1.5565 | WT |
| TG(16:0/22:5/18:1) | HMDB44453 | 0.019181908 | 0.67645 | 0.35331 | 1.1255 | WT |
| TG(14:0/18:2/14:0) [iso3] | HMDB42511 | 0.021069583 | 0.1514 | 1.0064 | 0.84351 | WT |
| PC(O-16:0/20:4) | HMDB13407 | 0.021212284 | 0.5914 | 0.52485 | 1.2804 | WT |
| TG(18:1/18:2/18:2)[iso3] | HMDB05461 | 0.02188706 | 0.60027 | 0.45845 | 1.2657 | WT |
| TG(16:0/22:5/16:0) [iso3] | HMDB44445 | 0.023122499 | 0.60027 | 0.46095 | 1.2662 | WT |
| DG(18:1/18:2/0:0) | HMDB07190 | 0.023860563 | 0.3561 | 0.74858 | 1.6983 | WT |
| ALA | HMDB29704 | 0.027755197 | 0.63176 | 0.41586 | 1.1162 | TG |
| TG(18:0/18:2/20:4)[iso6] | HMDB05412 | 0.030445202 | 0.67645 | 0.35549 | 1.1334 | WT |
| PC(16:0/20:4) | HMDB07982 | 0.033481958 | 0.60027 | 0.46508 | 0.92794 | WT |
| TG(16:0/22:5/18:2) [iso6] | HMDB44458 | 0.034453488 | 0.75389 | 0.27603 | 1.1304 | WT |
| PC(P-18:0/20:4) | HMDB11253 | 0.036743404 | 0.98824 | 0.009125 | 0.99726 | WT |
| TG(14:0/14:0/18:1) [iso3] | HMDB42071 | 0.036926684 | 0.67466 | 0.37473 | 0.92489 | WT |
| PC(18:0/22:6) | HMDB08057 | 0.037706136 | 0.070391 | 1.1784 | 0.27867 | TG |
| PI(16:0/20:4) | HMDB09789 | 0.03823482 | 0.04217 | 1.2645 | 1.9195 | WT |
| PE(18:0/18:2) | HMDB08994 | 0.040083484 | 0.10569 | 1.1068 | 9189.6 | WT |
| Cholesterol sulfate | HMDB00653 | 0.040692609 | 0.11065 | 1.0864 | 1.4855 | WT |
| PE(20:0/22:6) | HMDB09243 | 0.043501476 | 0.05081 | 1.2292 | 0.52915 | TG |
| PC(18:0/20:4) | HMDB08048 | 0.044487346 | 0.98824 | 0.011694 | 0.99633 | WT |
| LysoPE(18:0) | HMDB11130 | 0.044886472 | 0.049171 | 1.2396 | 1.4801 | WT |
| LysoPC(22:6) | HMDB10404 | 0.045975604 | 0.001165 | 1.6024 | 0.41706 | TG |
| TG(16:0/18:2/18:2)[iso3] | HMDB05390 | 0.047208921 | 0.81954 | 0.19965 | 1.1055 | WT |
| TG(16:0/16:1/18:2)[iso6] | HMDB05379 | 0.047572117 | 0.97056 | 0.060549 | 0.97574 | WT |
| LysoPE(18:2) | HMDB11477 | 0.047750431 | 0.37117 | 0.72716 | 1.7127 | WT |
| Cholesterol | HMDB00067 | 0.048975431 | 0.47186 | 0.51937 | 1.4483 | WT |
